# Impact of Age on the Treatment and Survival in Esophagogastric Cancer

**DOI:** 10.1245/s10434-022-13052-4

**Published:** 2023-01-17

**Authors:** Joonas H. Kauppila, Fredrik Mattsson, Jesper Lagergren

**Affiliations:** 1 Upper Gastrointestinal Surgery, Department of Molecular Medicine and Surgery, Karolinska Institutet, and Karolinska University Hospital, Stockholm, Sweden; 2 Surgery Research Unit, Medical Research Center, University of Oulu and Oulu University Hospital, Oulu, Finland; 3 School of Cancer and Pharmaceutical Sciences, King’s College London, and Guy’s and St Thomas’ NHS Foundation Trust, London, England

## Abstract

**Background:**

The age-specific risks of mortality for patients with esophagogastric cancer and their probability of surgical treatment are not well-known.

**Methods:**

This population-based, nationwide cohort study included all patients with esophageal or gastric (esophagogastric) cancer in Sweden between 1990 and 2013, with follow-up evaluation throughout 2018. Age at diagnosis (exposure) was categorized into nine 5-year groups. The main outcome was 5-year all-cause mortality. The secondary outcomes were 90-day all-cause mortality, 5-year disease-specific mortality, 5-year disease-specific mortality excluding 90-day all-cause mortality, and non-operation. For mortality outcomes, Cox regression provided hazard ratios (HRs) with 95% confidence intervals (95% CIs) adjusted for confounders. For non-operation, logistic regression provided odds ratios (ORs) with 95% CIs.

**Results:**

Among 28,725 patients, 11,207 (39.0%) underwent surgery. For those who underwent surgery, the HRs of 5-year all-cause mortality were stable before the ages of 65 to 69 years. After that, it gradually increased for patients 65 to 69 years old (HR, 1.13; 95% CI, 1.01–1.26), patients 75 to 79 years old (HR, 1.29; 95% CI, 1.56–1.44), and patients older than 85 years (HR, 1.84; 95% CI, 1.60–2.11) compared with those younger than 50 years. Analyses of age as a continuous variable, other mortality outcomes and stratification by comorbidity and tumor type showed similar results. The odds of non-operation increased for patients 75 to 79 years old (OR, 2.09 [95% CI, 1.84–2.94] for patients 80 to 84 years old and OR, 5.00 [95% CI, 4.31–5.78] for patients ≥85 years old or older), compared with those younger than 50 years.

**Conclusion:**

Older age, starting from 65 years, is associated with worse survival after surgery for esophagogastric cancer, and from 75 years with lower odds of surgical treatment.

**Supplementary Information:**

The online version contains supplementary material available at 10.1245/s10434-022-13052-4.

Esophageal and gastric (esophagogastric) cancers have many similarities including symptoms, diagnosis, treatment, follow-up findings, and prognosis. These tumors are among the most common cancers globally, and the overall 5-year survival is less than 20%.^1,2^ In 2020, these tumors caused more than 3.1 million deaths.^2^ The main curative treatment is surgical resection, often combined with perioperative chemotherapy.^3,4^

The surgical procedures for these tumors are extensive, often followed by serious complications and long-lasting morbidity. Except for tumor stage, comorbidity and age are important determinants of eligibility for curatively intended treatment.^3^

Regarding mortality, older age seems to be associated with higher mortality after surgery for esophageal cancer, at least in the short term,^5–7^ whereas older age may be associated with higher all-cause mortality after surgery for gastric cancer, but not with disease-specific mortality.^8^ Results from meta-analyses show a further need to assess the age at which the risk of mortality starts to increase for esophagogastric cancer patients.^9,10^

The peak in incidence of esophagogastric cancers is between 70 and 75 years in Sweden,^11,12^ whereas patients who undergo surgery are on the average 5 to 8 years younger than those not treated with surgery.^11,12^ The extent to which age, independent of the main prognostic factors, influences the selection of patients for curatively intended surgery is unclear.

This study aimed to clarify how age relates to survival after surgical treatment for esophagogastric cancer and to assess how age influences the selection of patients for surgical treatment in an unselected setting.

## Methods

### Design

This population-based and nationwide Swedish cohort study included all patients with a diagnosis of esophagogastric cancer (i.e., those with esophageal adenocarcinoma, esophageal squamous cell carcinoma, gastric cardia adenocarcinoma, or gastric non-cardia adenocarcinoma) in Sweden between 1990 and 2013, with follow-up evaluation for survival until 30 April 2018. Other tumor histologies and locations were excluded due to differences in treatment and prognosis. The study was approved by the Regional Ethical Review Board in Stockholm, Sweden.

### Data Collection

Patients with esophagogastric cancer (codes C15–C16 according to the International Classification of Diseases, version 10) were identified from the Swedish Cancer Registry, which has 98% nationwide completeness in the recording of these tumors.^13,14^ The Cancer Registry also provided information about tumor stage, but only for patients with a diagnosis from 2005 onward. The nationwide Swedish Patient Registry was used to identify patients who had undergone surgical resection (codes 2820–2829 and 4411-4435 from 1990 to 1997 and codes JCC00–JCC97 and JDC00–JDD96 from 1997 to 2013). The Registry has 99.6% positive predictive value for esophagectomy.^15^

The Patient Registry also provided information about patients’ age, sex, and comorbidity, as well as the hospitals’ annual volume of these surgical procedures. Comorbidity was defined and categorized using the well-validated and most updated version of the Charlson Comorbidity Index.^16^ Annual hospital volume was calculated as a 4-year moving average of annual resections. Mortality data were obtained from the nationwide Swedish Cause of Death Registry, which has 100% completeness for the date of death and at least 96% completeness for causes of death.^17^ The data from the different registries were linked and merged for each patient using the immutable personal identity number assigned to each Swedish resident at birth or immigration.^18^

### Exposure

The study exposure was patient age at the date of an esophagogastric cancer diagnosis. The date of birth was retrieved from the personal identity number. Patient age was categorized into nine groups (<50, 50–54, 55–59, 60–64, 65–69, 70–74, 75–79, 80–84, and ≥85 years of age) and also treated as a continuous variable. The purpose of this categorization was to balance between allowing a large number of 5-year age categories and preserving statistical power. In stratified analyses, age was categorized into three groups (<60, 60–74, and ≥75 years of age) instead of nine groups to preserve statistical power.

### Outcomes

Five outcomes were analyzed. The primary outcome was 5-year all-cause mortality. The secondary outcomes were 90-day all-cause mortality, 5-year disease-specific mortality, 5-year disease-specific mortality excluding 90-day all-cause mortality, and surgical resection (yes or no).

### Statistical Analysis

For the mortality outcomes, multivariable Cox regression was used to calculate hazard ratios (HRs) with 95% confidence intervals (CIs). The outcome of surgical resection was instead analyzed using logistic regression to obtain odds ratios (ORs) with 95% CIs. Except for an unadjusted (crude) model, a first multivariable model adjusted for five prognostic factors: (1) calendar year of diagnosis (continuous), (2) sex (male or female), (3) comorbidity (Charlson comorbidity index score 0, 1, 2, or ≥3), (4) tumor type (esophageal adenocarcinoma, esophageal squamous cell carcinoma, cardia adenocarcinoma, or non-cardia gastric adenocarcinoma), and (5) annual hospital volume of esophagogastric cancer surgery (in quartiles, i.e., four groups about equal in size). A second multivariable model further adjusted for tumor stage (0–1, 2, or 3–4), but because information on tumor stage was available only from 2005 onward and had high validity only for surgically treated patients, this model used only in a subgroup analysis of patients who underwent surgery between 2005 and 2013.

Analyses stratified by comorbidity and tumor type (categorized as described earlier) also were performed. The proportional hazards assumption was evaluated using log–log survival plots and by calculating the correlations between Schoenfeld residuals for a particular prognostic factor and ranking of individual failure time. The correlations were low, indicating that the proportional hazards assumption was met for all covariates. Because the rates of missing data were low, we performed a complete case analysis. All statistical analyses were conducted by an experienced biostatistician (F.M.), who followed an a priori specified study protocol. The statistical software SAS version 9.4 (SAS Institute, Gary, NC, USA) was used for all analyses.

## Results

### Patients

The 11,207 (39%) patients who underwent surgery were included in the analyses of mortality outcomes (Table 1), and all 28,725 patients with esophagogastric cancer were used for analyses of selection for surgery or no surgery (Table S1). The age distribution was similar across calendar time and sex, whereas the comorbidity scores and rates of gastric cancer diagnoses increased with older age.

**Table 1:** Characteristics of 11,207 patients who underwent surgery for esophagogastric cancer in Sweden between 1990 and 2013, categorized into nine age groups

Age (years)	<50 *n* (%)	50–54 *n* (%)	55–59 *n* (%)	60–64 *n* (%)	65–69 *n* (%)	70–74 *n* (%)	75–79 *n* (%)	80–84 *n* (%)	≥85 *n* (%)	Total *n* (%)
Total	679 (6.1)	657 (5.9)	981 (8.8)	1338 (11.9)	1731 (15.5)	2133 (19.0)	1957 (17.5)	1257 (11.2)	474 (4.2)	11207 (100)
Median year of diagnosis (IQR)	1999 (1994–2005)	1999 (1995–2006)	2001 (1995–2006)	2001 (1994–2007)	2000 (1994–2007)	1998 (1993–2004)	1998 (1993–2004)	1999 (1994–2005)	2000 (1995–2005)	1999 (1994–2005) *n*
Sex										
Male	418 (61.6)	465 (70.8)	692 (70.5)	947 (70.8)	1257 (72.6)	1448 (67.9)	1253 (64.0)	759 (60.4)	245 (51.7)	7484 (66.8)
Female	261 (38.4)	192 (29.2)	289 (29.5)	391 (29.3)	474 (27.4)	685 (32.1)	704 (36.0)	498 (39.6)	229 (48.3)	3723 (33.2)
Charlson Comorbidity Index										
0	548 (80.7)	513 (78.1)	750 (76.5)	906 (67.7)	1076 (62.2)	1212 (56.8)	1077 (55.0)	649 (51.6)	226 (47.7)	6957 (62.1)
1	119 (17.5)	122 (18.6)	197 (20.1)	346 (25.9)	529 (30.6)	694 (32.5)	626 (32.0)	426 (33.9)	188 (39.7)	3247 (29.0)
2	9 (1.3)	19 (2.9)	27 (2.8)	73 (5.5)	103 (6.0)	183 (8.6)	202 (10.3)	127 (10.1)	49 (6.2)	792 (7.1)
≥3	3 (0.4)	3 (0.5)	7 (0.7)	13 (1.0)	23 (1.3)	44 (2.1)	52 (2.7)	55 (4.4)	11 (2.3)	211 (1.9)
Tumor type										
EAC	60 (8.8)	86 (13.1)	128 (13.1)	191 (14.3)	212 12.3)	218 (10.2)	123 (6.3)	44 (3.5)	6 (1.3)	1068 (9.5)
ESCC	51 (7.5)	91 (13.9)	157 (16.0)	205 (15.3)	216 (12.5)	186 (8.7)	119 (6.1)	23 (1.8)	4 (0.8)	1052 (9.4)
Cardia GC	151 (22.2)	153 (23.3)	199 (20.3)	278 (20.8)	307 (17.7)	317 (14.9)	245 (12.5)	96 (7.6)	16 (3.4)	1762 (15.7)
Non-cardia GC	417 (61.4)	327 (49.8)	497 (50.7)	664 (49.6)	996 (57.5)	1412 (66.2)	1470 (75.1)	1094 (87.0)	448 (94.5)	7325 (65.4)
Annual hospital volume										
1st quartile	128 (18.9)	130 (19.8)	204 (20.8)	302 (22.6)	388 (22.4)	568 (26.6)	522 (26.7)	345 (27.5)	138 (5.1)	2725 (24.3)
2nd quartile	148 (21.8)	137 (20.9)	243 (24.8)	300 (22.4)	424 (24.5)	522 (24.5)	465 (23.8)	305 (23.3)	101 (21.3)	2645 (23.6)
3rd quartile	200 (29.5)	182 (27.7)	252 (25.7)	358 (26.8)	463 (26.8)	580 (27.2)	502 (25.7)	313 (24.9)	115 (24.3)	2965 (26.5)
4th quartile	203 (29.9)	208 (31.7)	282 (28.8)	378 (28.3)	456 (26.3)	463 (21.7)	468 (23.9)	294 (23.4)	120 (25.3)	2872 (25.6)

IQR, interquartile range; EAC, esophageal adenocarcinoma; ESCC, esophageal squamous cell carcinoma; Cardia GC, cardia gastric cancer; Non-cardia GC, non-cardia gastric cancer.

### Age and Risk of 5-Year All-Cause Mortality After Surgery

The adjusted HRs of 5-year all-cause mortality after surgery were stable in the younger age groups, then started to increase for the group 65 to 69 years old (HR, 1.13; 95% CI, 1.01–1.26) and further increased for each older age group (HR, 1.29 [95% CI, 1.56–1.44] for patients 75 to 79 years old and HR, 1.84 [95% CI, 1.60–2.11] for those ≥85 years old) compared with those younger than 50 years (Table 2). When age was analyzed as a continuous variable, the adjusted HRs of 5-year all-cause mortality increased by 1% (HR, 1.01; 95% CI, 1.01–1.02) for each year of age (Table 2).

**Table 2:** Age and risk of mortality in 11,207 patients who underwent surgery for esophagogastric cancer in Sweden between 1990 and 2013

Age (years)	<50 *n* (%)	50–54 *n* (%)	55–59 *n* (%)	60–64 *n* (%)	65–69 *n* (%)	70–74 *n* (%)	75–79 *n* (%)	80–84 *n* (%)	≥85 *n* (%)	Per year of age
Total	679 (6.1)	657 (5.9)	981 (8.8)	1338 (11.9)	1731 (15.5)	2133 (19.0)	1957 (17.5)	1257 (11.2)	474 (4.2)	11,207 (100.0)
5-Year all-cause mortality HR (95% CI)										
Crude	1 (reference)	1.07 (0.93–1.22)	1.00 (0.88–1.12)	1.14 (1.02–1.28)	1.18 (1.06–1.32)	1.25 (1.12–1.39)	1.34 (1.20–1.49)	1.58 (1.41–1.77)	1.79 (1.56–2.05)	1.01 (1.01–1.02)
Adjusted^a^	1 (reference)	1.04 (0.91–1.18)	0.97 (0.86–1.10)	1.09 (0.97–1.22)	1.13 (1.01–1.26)	1.18 (1.06–1.31)	1.29 (1.16 –1.44)	1.57 (1.40–1.75)	1.84 (1.60–2.11)	1.01 (1.01–1.02)
90-Day all-cause mortality HR (95% CI)										
Crude	1 (reference)	1.22 (0.74–2.00)	1.15 (0.73–18.3)	1.59 (1.04–2.42)	2.24 (1.51–3.33)	2.30 (1.56–3.40)	2.91 (1.98–4.28)	3.63 (2.45–5.37)	4.58 (3.01–6.98)	1.04 (1.03–1.05)
Adjusted^a^	1 (reference)	1.18 (0.72–1.94)	1.11 (0.70–1.75)	1.47 (0.97–2.24)	2.03 (1.36–3.02)	1.98 (1.34–2.92)	2.54 (1.72–3.75)	3.31 (2.23–4.92)	4.44 (2.90–6.81)	1.04 (1.03–1.04)
5-Year disease-specific mortality HR (95% CI)										
Crude	1 (reference)	1.07 (0.94–1.23)	0.99 (0.88–1.23)	1.11 (0.99–1.23)	1.15 (1.03–1.28)	1.19 (1.07–1.33)	1.24 (1.11–1.38)	1.41 (1.26–1.59)	1.59 (1.38–1.84)	1.01 (1.01–1.01)
Adjusted^a^	1 (reference)	1.05 (0.92–1.20)	0.98 (0.86–1.11)	1.07 (0.95–1.20)	1.11 (0.99–1.24)	1.13 (1.02–1.27)	1.21 (1.09–1.36)	1.43 (1.27–1.60)	1.66 (1.44–1.92)	1.01 (1.01–1.01)
5-Year disease-specific mortality excluding 90-day mortality HR (95% CI)										
Crude	1 (reference)	1.14 (0.99–1.31)	1.15 (1.01–1.31)	1.13 (1.00–1.28)	1.16 (1.04–1.30)	1.19 (1.06–1.34)	1.25 (1.11–1.41)	1.25 (1.11–1.41)	1.27 (1.09–1.49)	1.00 (1.00–1.01)
Adjusted^a^	1 (reference)	1.12 (0.98–1.29)	1.13 (1.00–1.29)	1.11 (0.99–1.26)	1.10 (0.98–1.24)	1.13 (1.01–1.26)	1.16 (1.04–1.31)	1.25 (1.10–1.41)	1.29 (1.10–1.51)	1.00 (1.00–1.01)

HR, hazard ratio; CI, confidence interval

^a^Adjusted for year of diagnosis, sex, comorbidity, tumor type, and annual hospital volume

In a sub-analysis of 2652 patients who underwent surgery between 2005 and 2013, with adjustment for pathologic tumor stage, the point estimates were similar to those in the main analysis (Table 3). The association between age and 5-year all-cause mortality was similar in analyses stratified by categories of time periods and tumor stages (Table 4 and Table S2). However, for the patients with at least three comorbidities, the risk increase over increasing age categories was greater than for the other comorbidity groups (Table 4 and Table S2). Similarly, the patients with esophageal adenocarcinoma seemed to have a slightly greater increase in relative mortality than those with esophageal squamous cell carcinoma, cardia adenocarcinoma, or gastric non-cardia adenocarcinoma (Table 4 and Table S2).

**Table 3:** Age and risk of mortality for 2652 patients who underwent surgery for esophagogastric cancer in Sweden between 2005 and 2013

Age (years)	<50 *n* (%)	50–54 *n* (%)	55–59 *n* (%)	60–64 *n* (%)	65–69 *n* (%)	70–74 *n* (%)	75–79 *n* (%)	80–84 *n* (%)	≥85 *n* (%)	Per year of age
	155 (5.8)	168 (6.3)	277 (10.4)	403 (15.2)	466 (17.6)	430 (16.2)	380 (14.3)	275 (10.4)	98 (3.7)	2652 (100.0)
5-Year all-cause mortality HR (95% CI)										
Crude	1 (reference)	1.00 (0.75–1.33)	1.00 (0.77–1.30)	1.25 (0.98–1.59)	1.19 (0.94–1.51)	1.32 (1.04–1.67)	1.37 (1.08–1.75)	1.83 (1.43–2.34)	2.39 (1.77–3.20)	1.02 (1.01–1.02)
Adjusted ^a^	1 (reference)	0.95 (0.71–1.27)	0.95 (0.73–1.23)	1.15 (0.90–1.46)	1.13 (0.89–1.43)	1.23 (0.97–1.57)	1.31 (1.03–1.68)	1.72 (1.34–2.22)	2.28 (1.69–3.09)	1.02 (1.01–1.02)
Adjusted ^b^	1 (reference)	0.93 (0.73–1.30)	1.00 (0.77–1.29)	1.20 (0.94–1.52)	1.17 (0.92–1.48)	1.25 (0.98–1.59)	1.45 (1.14–1.85)	1.88 (1.46–2.42)	2.62 (1.93–3.54)	1.02 (1.01–1.02)
90-Day all-cause mortality HR (95% CI)										
Crude	1 (reference)	1.11 (0.34–3.63)	0.90 (0.30–2.76)	1.48 (0.55–3.95)	1.63 (0.62–4.26)	2.52 (0.98–6.44)	3.08 (1.21–7.84)	4.06 (1.59–10.38)	5.19 (1.89–14.29)	1.05 (1.03–1.06)
Adjusted^a^	1 (reference)	1.05 (0.32–3.45)	0.84 (0.28–2.58)	1.28 (0.47–3.44)	1.42 (0.54–3.75)	1.95 (0.76–5.05)	2.54 (0.99–6.54)	2.91 (1.12–7.59)	4.04 (1.44–11.33)	1.04 (1.02–1.06)
Adjusted^b^	1 (reference)	1.06 (0.32–3.47)	0.85 (0.28–2.60)	1.27 (0.47–3.43)	1.41 (0.53–3.73)	1.91 (0.74–4.93)	2.62 (1.02–6.73)	3.00 (1.15–7.81)	4.51 (1.61–12.66)	1.04 (1.03–1.06)
5-Year disease-specific mortality HR (95% CI)										
Crude	1 (reference)	1.00 (0.75–1.35)	0.97 (0.74–1.27)	1.23 (0.96–1.58)	1.14 (0.89–1.46)	1.25 (0.98–1.60)	1.22 (0.95–1.57)	1.59 (1.23–2.07)	2.08 (1.52–2.85)	1.01 (1.01–1.02)
Adjusted^a^	1 (reference)	0.96 (0.71–1.29)	0.92 (0.70–1.20)	1.14 (0.89–1.46)	1.09 (0.85–1.39)	1.18 (0.92–1.51)	1.16 (0.90–1.50)	1.51 (1.16–1.97)	2.00 (1.45–2.76)	1.01 (1.01–1.02)
Adjusted^b^	1 (reference)	0.98 (0.73–1.32)	0.97 (0.74–1.27)	1.19 (0.93–1.53)	1.13 (0.88–1.45)	1.19 (0.93–1.53)	1.29 (1.00–1.67)	1.66 (1.27–2.16)	2.34 (1.69–3.22)	1.02 (1.01–1.02)
5-Year disease-specific mortality excluding 90-day mortality HR (95% CI)										
Crude	1 (reference)	1.21 (0.89–1.65)	1.38 (1.05–1.82)	1.30 (1.01–1.68)	1.17 (0.91–1.51)	1.30 (1.01–1.68)	1.33 (1.02–1.73)	1.46 (1.11–1.91)	1.60 (1.14–2.25)	1.01 (1.00–1.01)
Adjusted^a^	1 (reference)	1.20 (0.89–1.64)	1.28 (0.97–1.70)	1.24 (0.96–1.61)	1.16 (0.90–1.50)	1.25 (0.96–1.62)	1.30 (0.99–1.70)	1.47 (1.11–1.93)	1.65 (1.16–2.34)	1.01 (1.00–1.01)
Adjusted^b^	1 (reference)	1.17 (0.86–1.59)	1.23 (0.93–1.63)	1.22 (0.94–1.57)	1.13 (0.88–1.46)	1.17 (0.90–1.52)	1.30 (0.99–1.69)	1.47 (1.18–1.94)	1.67 (1.18–2.38)	1.01 (1.00–1.01)

HR, hazard ratio; CI, confidence interval

^a^Adjusted for year of diagnosis, sex, comorbidity, tumor type, and annual hospital volume

^b^Additionally adjusted for pathologic tumor stage

**Table 4:** Age and risk of 5-year all-cause mortality for 11,207 patients who underwent surgery for esophagogastric cancer in Sweden between 1990 and 2013, stratified by time period, comorbidity, and tumor type

Age (years)	<60	60–74	≥75	Per year of age
	HR (95% CI)	HR (95% CI)	HR (95% CI)	HR (95% CI)
Total				
Crude	1.00 (reference)	1.18 (1.11–1.25)	1.44 (1.36–1.54)	1.01 (1.01–1.02)
Adjusted^a^	1.00 (reference)	1.14 (1.07–1.21)	1.43 (1.34–1.53)	1.01 (1.01–1.02)
Time period				
1990–1996	1 (reference)	1.14 (1.03–1.26)	1.40 (1.26–1.55)	1.01 (1.01–1.02)
1997–2004	1 (reference)	1.12 (1.01–1.24)	1.36 (1.23–1.52)	1.01 (1.01–1.02)
2005–2008	1 (reference)	1.28 (1.09–1.51)	1.61 (1.36–1.92)	1.02 (1.01–1.02)
2009–2013	1 (reference)	1.06 (0.90–1.25)	1.47 (1.23–1.75)	1.02 (1.01–1.02)
Charlson Comorbidity Index				
0	1 (reference)	1.14 (1.06–1.23)	1.41 (1.31–1.53)	1.01 (1.01–1.01)
1	1 (reference)	1.13 (0.99–1.28)	1.46 (1.28–1.66)	1.02 (1.01–1.02)
2	1 (reference)	1.06 (0.77–1.47)	1.41 (1.02–1.94)	1.02 (1.01–1.03)
≥3	1 (reference)	1.80 (0.90–3.61)	1.66 (0.84–3.28)	1.01 (0.99–1.03)
Tumor type				
Esophageal adenocarcinoma	1 (reference)	1.25 (1.05–1.50)	1.65 (1.31–2.07)	1.02 (1.01–1.02)
Esophageal squamous cell carcinoma	1 (reference)	0.98 (0.83–1.14)	1.30 (1.04–1.62)	1.01 (1.00–1.02)
Cardia adenocarcinoma	1 (reference)	1.15 (1.01–1.31)	1.37 (1.18–1.60)	1.01 (1.00–1.01)
Gastric non-cardia adenocarcinoma	1 (reference)	1.16 (1.06–1.26)	1.45 (1.33–1.57)	1.02 (1.01–1.02)

HR, hazard ratio; CI, confidence interval

^a^Adjusted for year of diagnosis, sex, comorbidity, tumor type, and annual hospital volume

### Age and Risk of the Secondary Mortality Outcomes After Surgery

The adjusted HRs of 90-day mortality after surgery increased with older age and were significantly increased statistically for the group 65 to 69 years old or older (HR 2.03 [95% CI, 1.36–3.02] for those 65 to 69 years old and HR 4.44 [95% CI, 2.90–6.81] for those ≥85 years old) compared with those younger than 50 years (Table 2).

The adjusted HRs of 5-year disease-specific mortality were significantly increased statistically for the age group 70 to 74 years old or older (HR, 1.13 [95% CI, 1.02–1.27] for those 70 to 74 years old and HR 1.66 [95% CI, 1.44–1.92] for those ≥85 years old) compared with those younger than 50 years (Table 2).

The adjusted HRs of 5-year disease-specific mortality, excluding 90-day mortality, were increased from the group 70 to 74 years old or older (HR, 1.13 [95% CI, 1.01–1.26] for those 70 to 74 years old and HR, 1.29 [95% CI, 1.10–1.51] for those ≥85 years old) compared to those younger than 50 years (Table 2).

When age was analyzed as a continuous variable, the risk of all mortality outcomes increased with older age (Table 2). In a sub-analysis of patients treated with surgery between 2005 and 2013, including adjustment for pathologic tumor stage, the HRs were similar to those in the main analysis (Table 3).

### Age and Odds of Non-Operation

The non-operation rate was fairly similar in younger age groups but became increased from the group 75 to 79 years old (OR, 1.29; 95% CI, 1.14–1.46) and further increased in each older age group (Table 5). The adjusted ORs of non-operation were 2.09 (95% CI, 1.84–2.38) for those 80 to 84 years old and 5.00 (95% CI, 4.31–5.78) for those 85 years old or older compared to those younger than 50 years (Table 5).

**Table 5:** Age and odds of non-operation for 28,725 patients with esophagogastric cancer in Sweden between 1990 and 2013

Age (years)	<50 OR (95% CI)	50–54 OR (95% CI)	55–59 OR (95% CI)	60–64 OR (95% CI)	65–69 OR (95% CI)	70–74 OR (95% CI)	75–79 OR (95% CI)	80–84 OR (95% CI)	≥85 OR (95% CI)	Per year of age OR (95% CI)
*n* (%)	1407 (4.9)	1261 (4.4)	2007 (7.0)	2841 (9.9)	3857 (13.4)	4731 (16.5)	4986 (17.4)	4361 (15.2)	3274 (11.4)	28,725 (100.0)
Crude	1.00 (reference)	0.86 (0.74–1.00)	0.98 (0.85–1.12)	1.05 (0.92–1.19)	1.15 (1.01–1.30)	1.14 (1.01–1.28)	1.44 (1.28–1.63)	2.30 (2.04–2.61)	5.51 (4.78–6.35)	1.03 (1.03–1.04)
Adjusted^a^	1.00 (reference)	0.74 (0.63–0.87)	0.81 (0.71–0.94)	0.83 (0.73–0.95)	0.95 (0.84–1.07)	0.97 (0.86–1.10)	1.29 (1.14–1.46)	2.09 (1.84–2.38)	5.00 (4.31–5.78)	1.04 (1.03–1.04)

OR, odds ratio; CI, confidence interval

^a^Adjusted for year of diagnosis, sex, comorbidities, and tumor type

When age was analyzed as a continuous variable, the odds of non-operation increased by 4% with each year of age at diagnosis (adjusted OR, 1.04; 95% CI, 1.03–1.04; Table 5). In stratified analyses, the ORs were similar in comparisons of comorbidity groups, but the ORs of non-operation were increasingly high for older versus younger patients with a diagnosis during the later periods. The ORs also were higher for older versus younger patients with esophageal and cardia cancers versus patients with non-cardia gastric adenocarcinoma (Table S3).

## Discussion

In this study, higher patient age, starting from 65 to 69 years and older, was associated with gradually worse survival after surgery for esophagogastric cancer, and age 75 to 79 years or older was associated with a reduced likelihood of surgical treatment for these tumors. The associations between age and 5-year all-cause mortality remained after adjustment for prognostic factors and across categories of calendar period, comorbidity, tumor type, and tumor stage.

Several previous studies have examined patient age in relation to mortality in esophageal and gastric cancer. A meta-analysis of 25 observational studies and 12,104 patients who underwent esophagectomy for esophageal cancer suggested that older patients (older than 70 or 80 years) had increased in-hospital mortality (OR, 1.87; 95% CI, 1.54–2.26) and reduced 5-year survival (OR, 0.73; 95% CI, 0.62–0.87) compared with younger patients.^9^

Regarding surgically treated patients with gastric cancer, a meta-analysis of 21 mostly Asian studies and 18,179 patients found increased overall mortality for patients 80 years of age or older compared with younger patients (HR, 1.96; 95% CI, 1.65–2.27).^10^ However, there has been a lack of data to show the age at which the mortality starts to increase for esophagogastric cancer patients and how the risk develops across age groups.

The results of the current study suggest that the risk of 5-year all-cause mortality starts to increase at the age of 65 to 69 years, then continues to increase gradually with older age. This increase in mortality was more pronounced for the patients with multiple comorbidities and esophageal adenocarcinoma.

The finding that older age, after adjustment for other prognostic factors, reduces the likelihood of non-operation for esophagogastric cancers is not surprising considering the associations between age and general physical fitness, mental well-being, and comorbidity. Yet, surprisingly few studies have studied this question. An Irish study of 3165 patients indicated that patients 60 years old or older were far less likely to receive surgery for esophageal or gastric cardia cancer than those younger than 60 years, with increasing non-operation rates as age increased.^19^

A study of 5972 patients based on the Surveillance Epidemiology and End Results (SEER) database in the United States showed that patients 80 years old or older with non-metastatic gastric cancer were less likely to receive surgery than younger patients.^20^ However, these studies categorized age in wide intervals. In the current study, older age increased the risk of non-operation in a “dose-response manner,” starting from age 75 to 79 years and followed by a substantial increase for each older age group. This risk increase seemed to be greater during later time periods, for patients with multiple comorbidities, and for those with esophageal or cardia adenocarcinoma.

Aging brings challenges to the management of many esophagogastric cancer patients when clinicians are making decisions on whether to recommend the required extensive surgical resections or not. Careful individualized consideration is needed when frailty, malnutrition, comorbidity, and deteriorating organ functions argue against surgery because non-operation for these tumors usually leads to rapid deterioration and death. A common impression is that because older patients are more likely to experience postoperative problems, they may be more frequently undertreated, either by omission of treatment or by treatment with less extensive surgery and less effective methods.

On one hand, a randomized clinical trial suggested that elderly patients with gastric cancer more frequently undergo limited resections, leading to worse survival.^21^ On the other hand, outcomes for elderly patients undergoing esophagectomy for cancer equaling those for younger patients have been described.^22^ With increasingly older populations in many countries, critical decisions regarding patient age will be confronted more frequently.

Some future implications of this study may be considered. From a clinical point of view, age of 65 to 69 years should be part of the decision-making whether to recommend surgery or not, and increasingly so with older age. However, older age might introduce a risk of under-treatment.

From a research perspective, the current study indicates a need for studies examining factors influencing survival for different age groups, including the utility of objective assessment of fitness (e.g., exercise tests, stair-climbing tests, and spirometry).^23^ Such research may guide a more precise evaluation of the potential benefits and harm that surgery may bring to different age groups.

Among the methodologic strengths of this study were the population-based design with virtually complete inclusion and follow-up evaluation of the patients; the complete and reliable information regarding age (exposure), mortality, and surgery (outcomes); the main prognostic factors (confounders); and the large sample size. These advantages mitigated biases and facilitate generalizability.

The study also had limitations. Studies of age in relation to mortality might be susceptible to competing risk of death from causes other than cancer. The study accounted for this issue by also analyzing disease-specific mortality, and similar results regarding all-cause and disease-specific mortality argue against such influence. Confounding is a threat to most observational studies, which was reduced by adjusting for prognostic factors. However, confounding still could have existed due to other potentially prognostic factors (e.g., physical fitness, body mass index, smoking, alcohol overconsumption, and other lifestyle factors). However, confounding by these factors should have been reduced by the adjustment for the Charlson Comorbidity Index, which included diseases associated with these factors, and, again, the similar results for all-cause and disease-specific mortality argue against unknown or residual confounding.

Lack of reliable clinical stage for those not undergoing surgery may have led to confounding in analyses including non-operation patients. Chance errors cannot be ruled out. However, these were reduced by the large sample size, the reduction of age categories from nine to three in the stratified analyses, and the pre-defined protocol strictly followed in the analyses.

Systemic therapies for all studied tumor types have changed over time. Unfortunately, complete or reliable data on systemic therapies were not available from the registries. This was partly taken into account in the analyses showing no effect modification by calendar year for the association between age of diagnosis and mortality.

In conclusion, this large population-based cohort study indicated that increasing patient age from 65 to 69 years and gradually older age independently increases the risk of 5-year and shorter-term all-cause and disease-specific mortality in esophagogastric cancer after resection surgery. The study also showed that age from 75 to 79 years is associated with a lower rate of surgical treatment after adjustment for other prognostic factors, suggesting a level of under-treatment depending on age.

## Supplementary Information

Below is the link to the electronic supplementary material.

Supplementary file1 (DOCX 25 kb)
