# Supplementary material for: Impact of Age on the Treatment and Survival in Esophagogastric Cancer
Source: Ann Surg Oncol. 2023 Jan 17;30(5):2716–25. doi: 10.1245/s10434-022-13052-4 (PMC10085923; doi:10.1245/s10434-022-13052-4)
Supplement: Supplementary file 1 — Supplementary file1 (DOCX 25 kb) [file 10434_2022_13052_MOESM1_ESM.docx]

**Supplements**

Supplementary Table 1. Characteristics of patients with esophagogastric cancer in Sweden between 1990 and 2013.

Supplementary Table 2. Age and 5-year all-cause mortality of patients undergoing surgery for esophagogastric cancer in Sweden between 2005 and 2013 (n=2652) by age, stratified by comorbidity, tumor type, and tumor stage

Supplementary Table 3. Age and risk of non-operation for esophagogastric cancer patients in Sweden between 1990 and 2013 (n=28725), stratified by comorbidity and tumor type

**Supplementary Table 1. Characteristics of 28,725 patients with esophagogastric cancer in Sweden between 1990 and 2013, categorized into 9 age groups.**

|  | **<50 years** | **50-54 years** | **55-59 years** | **60-64 years** | **65-69 years** | **70-74 years** | **75-79 years** | **80-84 years** | **≥85 years** | **Total** |
| --- | --- | --- | --- | --- | --- | --- | --- | --- | --- | --- |
| **Total Number (%)** | 1407 (4.9) | 1261 (4.4) | 2007 (7.0) | 2841 (9.9) | 3857 (13.4) | 4731 (16.5) | 4986 (17.4) | 4361 (15.2) | 3274 (11.4) | 28725 |
| **Year of diagnosis, median [IQR]** | 2000 (1995-2006) | 2000 (1995-2007) | 2002 (1996-2007) | 2002 (1996-2008) | 2001 (1995-2008) | 1999 (1994-2006) | 2000 (1994-2006) | 2001 (1995-2006) | 2001 (1996-2007) | 2001 (1995 - 2007) |
| **Sex** |  |  |  |  |  |  |  |  |  |  |
| Male | 868 (61.7) | 897 (71.1) | 1428 (72.0) | 2046 (72.0) | 2779 (72.1) | 3264 (69.0) | 3227 (64.7) | 2644 (60.6) | 1780 (54.4) | 18933 (65.9) |
| Female | 539 (38.3) | 364 (28.9) | 579 (28.9) | 795 (28.0) | 1078 (27.9) | 1467 (31.0) | 1759 (35.3) | 1717 (39.4) | 1494 (45.6) | 9792 (34.1) |
| **Charlson comorbidity index** |  |  |  |  |  |  |  |  |  |  |
| 0 | 1072 (76.2) | 936 (74.2) | 1434 (71.5) | 1796 (63.2) | 2225 (57.7) | 2433 (51.4) | 2433 (48.8) | 1962 (45.0) | 1388 (42.4) | 15679 (54.6) |
| 1 | 295 (21.0) | 271 (21.5) | 475 (23.7) | 817 (28.8) | 1219 (31.6) | 1600 (33.8) | 1700 (34.1) | 1574 (36.1) | 1251 (38.2) | 9202 (32.0) |
| 2 | 32 (2.3) | 46 (3.7) | 75 (3.7) | 179 (6.3) | 312 (8.1) | 510 (10.8) | 657 (13.2) | 602 (13.8) | 471 (14.4) | 2884 (10.0) |
| 3 or more | 8 (0.6) | 8 (0.6) | 23 (1.2) | 49 (1.7) | 101 (2.6) | 188 (4.0) | 196 (3.9) | 223 (5.1) | 164 (5.0) | 960 (3.3) |
| **Tumor type** |  |  |  |  |  |  |  |  |  |  |
| EAC | 151 (10.7) | 206 (16.3) | 308 (15.4) | 468 (16.5) | 568 (14.7) | 589 (12.5) | 509 (10.2) | 473 (10.9) | 351 (10.7) | 3623 (12.6) |
| ESCC | 137 (9.7) | 213 (16.9) | 395 (19.7) | 578 (20.3) | 687 (17.8) | 780 (16.5) | 683 (13.7) | 495 (11.4) | 351 (10.7) | 4319 (15.0) |
| Cardia | 294 (20.9) | 265 (21.0) | 383 (19.1) | 546 (19.2) | 633 (16.4) | 715 (15.1) | 706 (14.1) | 532 (12.2) | 389 (11.9) | 4463 (15.5) |
| NCGC | 825 (58.6) | 577 (45.8) | 921 (45.9) | 1249 (44.0) | 1969 (51.1) | 2647 (56.0) | 3088 (61.9) | 2861 (65.6) | 2183 (66.7) | 16320 (56.8) |
| **Surgical treatment** |  |  |  |  |  |  |  |  |  |  |
| Yes | 679 (48.3) | 657 (52.1) | 981 (48.9) | 1338 (47.1) | 1731 (44.9) | 2133 (45.1) | 1957 (39.2) | 1257 (28.8) | 474 (14.5) | 11207 (39.0) |
| No | 728 (51.7) | 604 (47.9) | 1026 (51.1) | 1503 (52.9) | 2126 (55.1) | 2598 (54.9) | 3029 (60.8) | 3104 (71.2) | 2800 (85.5) | 17518 (61.0) |

Abbreviations: IQR, Interquartile range; EAC, esophageal adenocarcinoma; ESCC, esophageal squamous cell carcinoma; Cardia GC, cardia gastric cancer; Non-cardia GC, Non-cardia gastric cancer.

**Supplementary Table 2.**  **Age and 5-year all-cause mortality in 2,652 patients who underwent surgery for esophagogastric cancer in Sweden between 2005 and 2013, stratified by time period, comorbidity, tumor type, and tumor stage.**

|  | **<60 years**  **Hazard ratio (95% confidence interval)** | **60-74 years**  **Hazard ratio (95% confidence interval)** | **≥75 years**  **Hazard ratio (95% confidence interval)** | **Per year of age**  **Hazard ratio (95% confidence interval)** |
| --- | --- | --- | --- | --- |
| **Total** |  |  |  |  |
| Crude | 1 (reference) | 1.25 (1.10-1.42) | 1.64 (1.43-1.88) | 1.02 (1.01-1.02) |
| Model 2* | 1 (reference) | 1.21 (1.07-1.38) | 1.60 (1.39-1.85) | 1.02 (1.01-1.02) |
| Model 3** | 1 (reference) | 1.21 (1.07-1.38) | 1.71 (1.49-1.98) | 1.02 (1.01-1.02) |
|  |  |  |  |  |
| **Time period** |  |  |  |  |
| 2005-2008 | 1 (reference) | 1.31 (1.09-1.57) | 1.81 (1.49-2.21) | 1.02 (1.01-1.03) |
| 2009-2013 | 1 (reference) | 1.12 (0.94-1.35) | 1.61 (1.33-1.96) | 1.02 (1.01-1.03) |
|  |  |  |  |  |
| **Charlson comorbidity index** | |  |  |  |
| 0 | 1 (reference) | 1.19 (1.03-1.38) | 1.67 (1.41-1.98) | 1.02 (1.01-1.02) |
| 1 | 1 (reference) | 1.21 (0.91-1.62) | 1.86 (1.38-2.50) | 1.03 (1.02-1.04) |
| 2 | 1 (reference) | 1.28 (0.62-2.66) | 1.53 (0.74-3.18) | 1.01 (0.99-1.03) |
| ≥3 | 1 (reference) | 2.51 (0.86-7.31) | 2.99 (1.04-8.65) | 1.04 (1.01-1.07) |
|  |  |  |  |  |
| **Tumor type** |  |  |  |  |
| Esophageal adenocarcinoma | 1 (reference) | 1.39 (1.02-1.89) | 2.39 (1.62-3.53) | 1.02 (1.01-1.04) |
| Esophageal squamous cell carcinoma | 1 (reference) | 0.87 (0.62-1.24) | 1.30 (0.80-2.11) | 1.01 (0.99-1.03) |
| Cardia adenocarcinoma | 1 (reference) | 1.41 (1.09-1.82) | 1.65 (1.20-2.26) | 1.01 (1.01-1.03) |
| Gastric non-cardia adenocarcinoma | 1 (reference) | 1.16 (0.96-1.41) | 1.66 (1.37-2.01) | 1.02 (1.01-1.03) |
|  |  |  |  |  |
| **Pathological tumor stage** | |  |  |  |
| 0-I | 1 (reference) | 1.07 (0.76-1.51) | 1.91 (1.37-2.67) | 1.03 (1.02-1.04) |
| II | 1 (reference) | 1.26 (1.00-1.61) | 1.51 (1.16-1.97) | 1.02 (1.01-1.02) |
| III-IV | 1 (reference) | 1.22 (1.03-1.44) | 1.74 (1.44-2.10) | 1.02 (1.01-1.02) |

*Adjusted for year of diagnosis, sex, comorbidity, tumor type, and annual hospital volume.

**Additionally adjusted for tumor stage.

**Supplementary Table 3.** **Age and odds of non-operation in 28,725 esophagogastric cancer patients in Sweden between 1990 and 2013, stratified by time period, comorbidity, and tumor type.**

|  | **<60 years**  **Odds ratio (95% confidence interval)** | **60-74 years**  **Odds ratio (95% confidence interval)** | ≥**75 years**  **Odds ratio (95% confidence interval)** | **Per year of age**  **Odds ratio (95% confidence interval)** |
| --- | --- | --- | --- | --- |
| **Total** |  |  |  |  |
| Crude | 1 (Reference) | 1.18 (1.10-1.26) | 2.38 (2.22-2.55) | 1.03 (1.03-1.04) |
| Adjusted* | 1 (Reference) | 1.09 (1.02-1.17) | 2.38 (2.22-2.57) | 1.04 (1.03-1.04) |
|  |  |  |  |  |
| **Time period** |  |  |  |  |
| 1990-1996 | 1 (reference) | 0.87 (0.79-0.95) | 1.83 (1.68-2.01) | 1.03 (1.03-1.03) |
| 1997-2004 | 1 (reference) | 1.07 (0.98-1.17) | 2.37 (2.17-2.59) | 1.04 (1.03-1.04) |
| 2005-2008 | 1 (reference) | 1.20 (1.06-1.34) | 2.96 (2.62-3.35) | 1.04 (1.03-1.04) |
| 2009-2013 | 1 (reference) | 1.55 (1.39-1.72) | 3.15 (2.80-3.55) | 1.04 (1.04-1.04) |
| **Charlson comorbidity index** | |  |  |  |
| 0 | 1 (Reference) | 1.14 (1.04-1.24) | 2.47 (2.26-2.70) | 1.03 (1.03-1.04) |
| 1 | 1 (Reference) | 0.94 (0.82-1.09) | 2.14 (1.86-2.47) | 1.04 (1.03-1.04) |
| 2 | 1 (Reference) | 1.11 (0.77-1.60) | 2.47 (1.72-3.53) | 1.05 (1.04-1.06) |
| 3 or more | 1 (Reference) | 2.15 (1.04-4.46) | 2.90 (1.42-5.91) | 1.05 (1.03-1.07) |
| **Tumor type** |  |  |  |  |
| Esophageal adenocarcinoma | 1 (Reference) | 1.04 (0.86-1.25) | 4.08 (3.26-5.10) | 1.05 (1.04-1.06) |
| Esophageal squamous cell carcinoma | 1 (Reference) | 1.46 (1.23-1.75) | 5.68 (4.53-7.12) | 1.06 (1.06-1.07) |
| Cardia adenocarcinoma | 1 (Reference) | 1.15 (0.98-1.34) | 3.63 (3.04-4.33) | 1.05 (1.04-1.05) |
| Gastric non-cardia adenocarcinoma | 1 (Reference) | 0.98 (0.89-1.08) | 1.72 (1.57-1.89) | 1.03 (1.02-1.03) |

*Adjusted for year of diagnosis, sex, comorbidity, and tumor type.
